# Prostate Cancer Screening: A Brief Tool to Incorporate Patient Preferences in a Clinical Encounter

**DOI:** 10.3389/fonc.2016.00235

**Published:** 2016-11-04

**Authors:** Anita D. Misra-Hebert, Michael W. Kattan

**Affiliations:** ^1^Department of Internal Medicine, Center for Value-Based Care Research, Medicine Institute, Cleveland Clinic, Cleveland, OH, USA; ^2^Department of Quantitative Health Sciences, Lerner Research Institute, Cleveland Clinic, Cleveland, OH, USA

**Keywords:** shared decision making, cancer screening, prostate cancer diagnosis, primary health care, screening tools

## The Problem

Conflicting results of two major randomized trials on prostate cancer mortality (1, 2) have led to evolving guidelines for clinicians regarding prostate cancer screening (3–6). While the European Randomised Study of Screening for Prostate Cancer (ERSPC) showed that one prostate cancer death could be prevented per 781 men screened, the Prostate, Lung, Colorectal, and Ovarian (PLCO) Cancer Screening Trial showed no mortality benefit at 13 years of follow-up (1, 2). Because of the question of mortality benefit and the possible harms of screening, the United States Preventive Services Task Force (USPSTF) initially recommended in 2008 against screening men over age 75 for prostate cancer (3) then in 2012 against screening for prostate cancer at any age (4). However, it is most recently recommended in 2013 by both the American College of Physicians (ACP) and the American Urologic Association (AUA) that screening for prostate cancer occurs through a process of shared decision making (5, 6). While it is not recommended to screen men for prostate cancer at age 70 or above, or men with a life expectancy of less than 10–15 years, for men with average risk of developing prostate cancer, these shared decisions should occur between physicians and patients for men starting at age 50. For those men with high risk of developing prostate cancer, including men with a family history of prostate cancer or African-American men, these conversations could start as early as age 40 (5, 6).

Most of these shared discussions will initially occur between men and their primary care physician. Previous reports of decision aids for prostate cancer screening have shown improvement in patient knowledge and in decisional conflict as well as satisfaction with the decision (7). However, it is also known that patients may be more likely to be aware of the benefits of screening decisions and not the harms, and may ultimately rely on the physician’s recommendation, and it is not clear if the use of decision support alters prostate-specific antigen (PSA) testing behavior (8). Indeed, the rates of PSA testing were reduced in older men after the initial USPSTF recommendations (9–11) and in men over age 50 after the 2012 recommendations (12). However, is it not known to what extent shared decision making contributed to the decreased screening rates observed, whether shared decision making is occurring more frequently since the ACP and AUA recommendations were released, or to what degree patient or provider factors have contributed to the decline in screening rates.

Barriers to shared decision making regarding prostate cancer screening include lack of time for discussions as well as physician and patient factors (13–18). Patients may address an average of 25 problems during some chronic care and primary care visits (19), and cancer screening discussions may not be prioritized when management of multiple chronic diseases is also needed during the same office visit. More importantly, primary care physicians do not readily have access to tools that can promote shared decision making for prostate cancer screening. Elwyn et al. have proposed a model of “choice, option, and decision talk” to be used for shared decision making conversations in the clinical encounter (20). For a complex decision, such as prostate cancer screening, we propose the use of a simple tool in a routine outpatient encounter to begin this discussion, to accomplish the first two steps of this model: (1) make the patient aware that he has a choice and (2) begin to consider the options. We suggest that the decision is discussed after the patient has had appropriate time to consider the choice and options. This step may occur within the same visit or at a later time.

## Explanation of Constructs

We propose shared decision making for prostate cancer in a primary care encounter is based upon nine constructs incorporating risk for prostate cancer and patient preferences.

These constructs can provide a framework for the physician–patient discussion of choice and options regarding prostate cancer screening and help the patient to understand the relative contribution of each of these constructs to the ultimate choice of “Yes, you may benefit from being screened with the PSA” vs. “No, you may not benefit from being screened with the PSA.”

### Constructs 1–5: Risk for Developing Prostate Cancer

#### Age

The risk for developing prostate cancer increases with age. The median age at diagnosis is 66 (21). However, screening at age 70 or above is not recommended (5, 6), based upon life expectancy (22).

#### Family History of Prostate Cancer

A family history of prostate cancer increases the relative risk of prostate cancer in a man with a first-degree relative with prostate cancer to 2.48, with a higher risk in men younger than age 65, or if the man’s brother had prostate cancer, and greater risk if more than one first-degree relative was affected (23, 24).

#### High vs. Low Risk Racial/Ethnic Group

African-American men have an increased risk of developing prostate cancer compared to Caucasian men, while men from Asian and Native American or Alaskan native backgrounds have a lower risk. Hispanic ethnicity is also associated with a lower risk of developing prostate cancer (21).

#### History of Previous Digital Rectal Exam

Data from the Prostate Cancer Prevention Trial suggest that the odds ratio for having prostate cancer is 2.47 if a man has an abnormal digital rectal exam (DRE) (25) but the sensitivity of the DRE is low. It is possible that primary physicians will not routinely have performed a DRE for risk stratification.

#### History of Previous Prostate-Specific Antigen Test

Risk of prostate cancer is predicted by PSA level (25) with recent recommendations suggesting different approaches to men using PSA cut off levels of <1, 1–3, and >3 ng/ml (26).

### Constructs 6–9: Patient Self-Reported Health Status/Preferences

#### Health Status/Comorbidities

We propose that a patient’s self-reported rating of quality of life is incorporated into the decision to offer screening for prostate cancer. While this initial tool does not quantitatively address comorbidities, we propose that clinicians will make an assessment of comorbidities with patients during the screening discussion after reviewing the patient’s answer to this question on the tool.

#### Importance of Urinary Symptoms

Urinary symptoms are a known side effect of prostate cancer treatment. Up to 14% of men can experience urinary incontinence 5 years after radical prostatectomy for treatment of prostate cancer (27–29). Assessment of the importance of maintaining urinary function should be performed as part of the prostate cancer screening discussion.

#### Importance of Maintaining Sexual Function

Erectile dysfunction is also a known side effect of radical prostatectomy (27–29) and can affect 50% of men undergoing treatment, with slightly lower rates after radiotherapy (30, 31).

#### Anxiety about Developing Prostate Cancer

Anxiety regarding prostate cancer may be a major driver in the screening decision (32, 33), and this issue should be addressed prior to making a choice to proceed with screening.

## A Brief Tool

While we understand that the decision for prostate cancer screening is complex, we also appreciate the limited time available to discuss this topic in a routine clinical encounter and the proposed tool is meant to begin a discussion that incorporates patient preferences and is not meant to be comprehensive. We propose a tool that can be given to patient within the clinical encounter (Table 1). Questions 1–5 address demographic or past medical history indicators of prostate cancer risk, while Questions 6–9 focus on patient preference. While we do not propose a specific “cut off” score to prompt a decision to screen for prostate cancer, we have suggested assigned points to answer options based upon our available data for each of our constructs.

**Table 1:** **Proposed screening tool and interpretation based upon constructs**.

Question	Answer Options with suggested scoring
1.What is your age?	≥70 (0)
	65–69 (3)
	55–64 (2)
	40–54 (1)
	<40 (0)
2.What is your race/ethnicity? Select all that apply	White/Caucasian (1)
	Black/African-American (3)
	Asian/Pacific Islander (0)
	Native American/Alaskan Native (0)
	Hispanic (0)
3.Did any of your family members have prostate cancer? More than one answer may apply	My father had prostate cancer (1)
	My brother had prostate cancer (2)
	More than one family member had prostate cancer (1) please consider only those family members related by birth, not by marriage only (skip if both father and brother had prostate cancer)
	I do not have a family history of prostate cancer or do not know about my family history of prostate cancer (0)
4.If you have had a digital rectal exam, what was the result?	No previous digital rectal exam (1)
	Normal digital rectal exam (0)
	Abnormal digital rectal exam (3)
5.If you have had previous prostate-specific antigen (PSA) level what was the result?	No previous PSA (1)
	PSA < 1.0 (0)
	PSA 1.0–3.0 (2)
	PSA > 3.0 (3)
6.How would you rate your health?	My health is excellent (3)
	My health is very good (2)
	My health is good (1)
	My health is poor (0)
7.If you were to have problems with incontinence (leakage) of urine, how bothersome would this be for you?	Not bothersome (3)
	Somewhat bothersome (2)
	Very bothersome (1)
	Extremely bothersome (0)
8.How important is your sex life and maintaining ability to have an erection?	Not important (3)
	Somewhat important (2)
	Very important (1)
	Extremely important (0)
9.Are you concerned about having or developing prostate cancer?	No, I am not concerned about having or developing prostate cancer (0)
	I am a little concerned I may have or may develop prostate cancer (1)
	I am very concerned I may have or may develop prostate cancer (2)
	I am extremely concerned I may have or may develop prostate cancer (3)

**Yes, you may benefit from being screened with the PSA if higher scores:**	**No, you may not benefit from being screened with the PSA if lower scores:**

Younger age, treating prostate cancer may have more benefits than risks	Older age, treating prostate cancer may have more risks than benefits
Extensive family history of prostate cancer	No family history of prostate cancer
Higher risk racial group	Lower risk racial group
Abnormal digital rectal exam or no previous digital rectal exam	Normal previous digital rectal exam
Previous PSA in higher risk range or no Previous PSA	Previous PSA in lower risk range
Excellent health status, life expectancy not reduced related to comorbid conditions	Poor health status, life expectancy reduced related to comorbid conditions
Urinary incontinence would not be bothersome	Urinary incontinence would be extremely bothersome
Sex life is not important	Sex life is extremely important
Extremely concerned about having or developing prostate cancer	Not concerned about having or developing prostate cancer

## Discussion

It is extraordinarily difficult to use a tool like this precisely to make a screening decision due to the enormous complexity of the decision. However, we think this tool could be useful as a guide for physicians to identify men who may benefit from further discussion vs. those who may not. Extreme values on this tool may indicate decisions on whether to screen. Scores toward the center values may indicate those who will need further thought and discussion, and possibly extensive shared decision making. Ideally, a physician would introduce this tool during an office visit with a phrase, such as “I would like to make you aware of the option you have to choose to have a screening test to look for prostate cancer. Some men may choose this test while others may not based upon their preferences. I have a few questions you can answer to help think about this option. I can then provide more information for you and we can discuss this at your next visit.” An alternative would be to have a patient fill out the tool prior to an office visit with a plan to discuss the results at the time of the visit. Testing of this tool in busy primary care clinical settings is underway, and the tool will be refined with continued feedback from clinicians and patients.

## Author Contributions

AM-H and MK contributed equally to the concept and design of this opinion piece. Both the authors have reviewed the final version of the manuscript being submitted.

## Conflict of Interest Statement

The authors declare that the research was conducted in the absence of any commercial or financial relationships that could be construed as a potential conflict of interest.
